# MicroRNA Biomarkers for Infectious Diseases: From Basic Research to Biosensing

**DOI:** 10.3389/fmicb.2020.01197

**Published:** 2020-06-03

**Authors:** Leon Tribolet, Emily Kerr, Christopher Cowled, Andrew G. D. Bean, Cameron R. Stewart, Megan Dearnley, Ryan J. Farr

**Affiliations:** ^1^Health and Biosecurity, Australian Animal Health Laboratory, Commonwealth Scientific and Industrial Research Organisation (CSIRO), Geelong, VIC, Australia; ^2^Institute for Frontier Materials, Deakin University, Geelong, VIC, Australia; ^3^Diagnostics, Surveillance and Response (DSR), Australian Animal Health Laboratory, Commonwealth Scientific and Industrial Research Organisation (CSIRO), Geelong, VIC, Australia

**Keywords:** microRNAs, infectious disease, diagnostics, biomarkers, personalized medicine

## Abstract

In the pursuit of improved diagnostic tests for infectious diseases, several classes of molecules have been scrutinized as prospective biomarkers. Small (18–22 nucleotide), non-coding RNA transcripts called microRNAs (miRNAs) have emerged as promising candidates with extensive diagnostic potential, due to their role in numerous diseases, previously established methods for quantitation and their stability within biofluids. Despite efforts to identify, characterize and apply miRNA signatures as diagnostic markers in a range of non-infectious diseases, their application in infectious disease has advanced relatively slowly. Here, we outline the benefits that miRNA biomarkers offer to the diagnosis, management, and treatment of infectious diseases. Investigation of these novel biomarkers could advance the use of personalized medicine in infectious disease treatment, which raises important considerations for validating their use as diagnostic or prognostic markers. Finally, we discuss new and emerging miRNA detection platforms, with a focus on rapid, point-of-care testing, to evaluate the benefits and obstacles of miRNA biomarkers for infectious disease.

## Infectious Disease Diagnostics: Current Outlook

Infectious diseases account for a large proportion of morbidity and 15% of total global mortality (WHO, 2018a). Due to the ease of global travel and potential for global health epidemics, there is a critical need for the early diagnosis of infected individuals to assist in containing the spread of disease. For many diseases early intervention and expedient treatment is required for a favorable prognosis. Sometimes clinical presentation or case history is sufficiently diagnostic, however, choosing an appropriate course of treatment often relies upon the outcome of laboratory-based diagnostic testing. The clinical diagnostic landscape for the detection of infectious diseases is diverse and includes either looking for the pathogen or looking for the host’s response to the pathogen. Conventional laboratory tests include *in vitro* culture and isolation, protein-based assays (for example ELISA and serology), microscopy (histological, pathological, and morphological assays), mass spectrometry (Kullolli et al., 2014) and molecular diagnostics which uses nucleic acid-based assays (such as quantitative (q)PCR and sequencing). Many of these methods require a substantial amount of time to perform and are reliant on intensive sample preparation, expert users, and suffer from technical limitations.

Diagnostic tests might perform within acceptance criteria yet give the wrong answer, resulting in either a false positive or false negative. This can be due to low sensitivity, low specificity, and/or not collecting a representative sample from the patient. In some cases, infections can be localized to specific cell types or tissues and a systemic sample, such as blood, may not contain detectable levels of the pathogen required for detection. In contrast, antibodies may be easily detected, however, antibody responses may take weeks to months to manifest, with the specificity of these responses sometimes hampered by cross-reactivity (Lanciotti et al., 2008). Antibody testing can be suitable for population level disease testing and analysis, to monitor epidemic/pandemic spread and to ascertain which patients have had prior exposure to a specific pathogen. Additionally, certain viruses, including rabies (Ito et al., 2016) and cytomegalovirus (Patro, 2019), efficiently modulate or evade the immune response, further complicating their detection. Tests for some pathogens are also ineffective during the early phases of infection. For example, the gold-standard diagnostic test for rabies (lyssavirus) infection is a fluorescent antibody test that can only be performed on post-mortem brain tissue. Other classes of diagnostic molecules include other nucleic acids such as messenger RNA (mRNA) for the detection of urothelial carcinoma (Urquidi et al., 2016), proteins such as procalcitonin (PCT) interleukin-6 (IL-6) and C-reactive protein (CRP) for sepsis (Lai et al., 2020) the presence of metabolites such as creatinine in serum or urea for renal disease (Stevens et al., 2006) or even presence of volatile compounds in breath analytics are being tested as cancer diagnostics (Markar et al., 2016).

In the search for new diagnostic biomarkers to circumvent these issues, many different classes of molecules have been studied. Amongst the most promising are microRNAs (miRNAs), small (18–22nt) non-coding RNA molecules found within all bodily fluids and tissues and most cell types (Halushka et al., 2018), which play an essential role in post-transcriptional regulation of gene expression. There are approximately 2,600 miRNAs in the human genome thus far according to the online miRNA repository, miRbase (version 22^1^) and approximately 2,000 miRNAs in circulation (Juzenas et al., 2017). MicroRNAs are named with the miR prefix followed by an identifying number. If there are extremely similar sequences, additional suffixes (letters or numbers) are provided. Older nomenclature can also refer to a miRNA as the guide (used to identify target mRNA) or passenger (denoted with a ‘^∗^’ suffix) miRNA. As more studies demonstrated that both strands can be functional (Jin et al., 2015; Gao et al., 2020), this was replaced with the use of -3p and -5p suffixes, denoting the 3′ or 5′ end of the miRNA precursor. For an in-depth review of miRNA biogenesis and function, please see Saliminejad et al. (2019).

## The Potential for miRNAs to Improve Disease Outcomes

In 2002, less than a decade after the discovery of miRNAs (Lee et al., 1993), the application of miRNAs as disease biomarkers was first explored. Calin et al. (2002) showed that miRNA expression patterns were altered in patients suffering from chronic lymphocytic leukemia. Shortly after this, miRNA expression was found to change during tumorigenesis (Michael et al., 2003), and could be successfully used to classify multiple human cancers (Lu et al., 2005). Since then, studies have examined the potential of miRNAs as biomarkers of diabetes (Farr et al., 2013, 2015a,b), Alzheimer’s Disease (Lugli et al., 2015), and numerous other non-infectious conditions. Meta-analyses of multiple cancer studies strongly support a role for miRNAs as diagnostic, providing the ability to identify a disease (Zhi et al., 2015) or prognostic, identifying the likelihood of developing specific disease outcomes (Schmitz et al., 2016) biomarkers. Additionally, miRNAs may extend the clinical utility of current proteins or metabolite-based tests. For instance, a recent paper demonstrated that miR-29a and miR-335 in combination with matrix metalloprotease protein-2 (MMP2), proved to be a superior diagnostic in breast cancer to the current carcinoembryonic antigen (CEA) and cancer antigen 15-3 (CA 15-3) tests that are widely used (Ali Ahmed et al., 2020).

Recently, several reports describe changes in circulating miRNAs in response to infectious diseases, raising the possibility for a new diagnostic tool against infections. MicroRNAs have been identified as potential biomarkers of infections caused by a range of pathogens, including Hendra virus (Stewart et al., 2013), HIV (Biswas et al., 2019), tuberculosis (Zhang et al., 2013), malaria (Li et al., 2018), including differentiating complicated and uncomplicated P. vivax malaria (Kaur et al., 2018) and Ebola (Duy et al., 2016). In some studies, changes in miRNA profiles were observed early in disease onset, before the pathogen could be directly detected and prior to the onset of seroconversion (Stewart et al., 2013; Biswas et al., 2019). MicroRNAs have also been implicated in influenza infections (Scheller et al., 2019) and rhinoviruses (Hasegawa et al., 2018). Therefore, the potential for miRNA diagnostics with other respiratory viruses, such as the recent severe acute respiratory syndrome coronavirus 2 (SARS-CoV-2) outbreak, is not to be understated. An identified and validated COVID-19 miRNA signature could provide differential diagnosis to identify COVID-19 infections from other infections with similar presenting symptoms such as influenza, rhinoviruses or other coronaviruses. Additionally, as mentioned above miRNAs have prognostic potential, to identify the likelihood of severe vs. mild disease outcomes and also to identify asymptomatic infections (Hou et al., 2017), which are problematic in epidemic or pandemic spread and disease tracking.

The potential utility of these molecules as diagnostic biomarkers is enhanced by their presence and stability in a wide range of biological fluids, including peripheral circulation. They show remarkable stability despite multiple freeze-thaw cycles or extreme pH (Chen et al., 2008) and are routinely measured via next generation sequencing (NGS) or qPCR (Farr et al., 2015a). Despite this, there are many infectious diseases for which miRNA biomarkers would be inappropriate, ineffectual, or highly difficult to implement in clinical practice. For example, short-term, self-limiting infections that require minimal intervention (apart from alleviating symptoms), such as the common cold or gastroenteritis, would not benefit from the development of these biomarkers. Others, particularly those with significant impact, currently sub-par diagnostics, or long subclinical phases would benefit greatly by the application of validated miRNA diagnostics. Therefore, it is important to understand the disease of interest and how miRNA biomarkers would be best applied before embarking on miRNA biomarker discovery.

### Advantages of miRNA Biomarkers I: Early Detection

**FIGURE 1:**
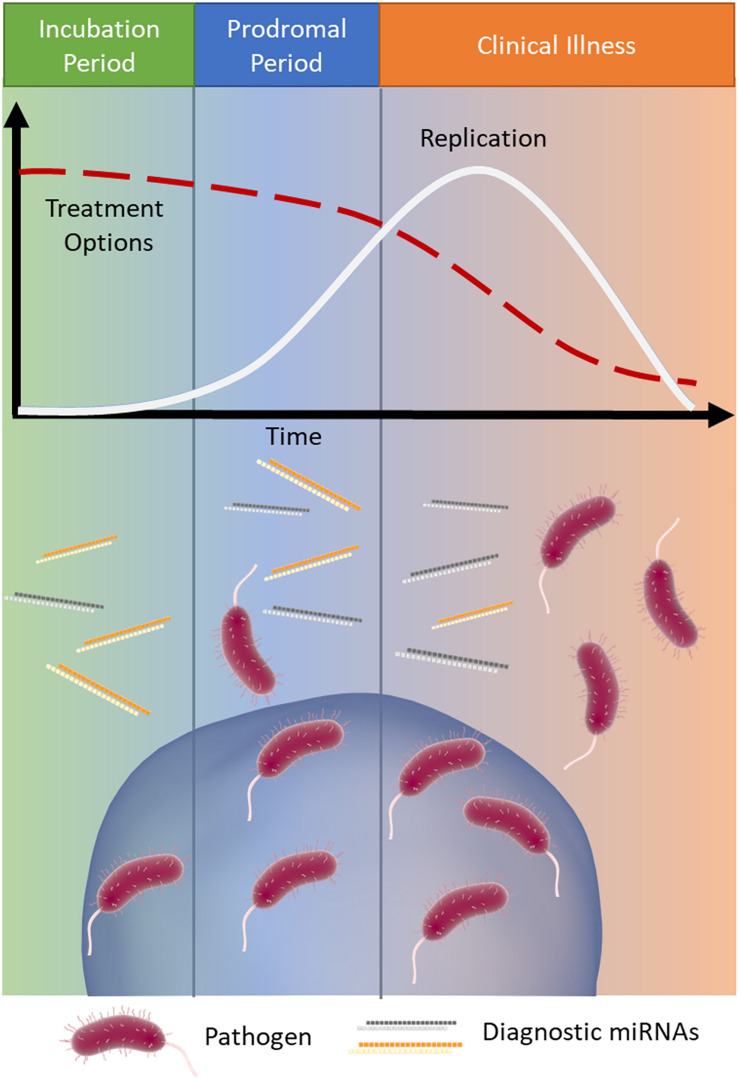
MicroRNA signatures during infection. Infections typically progress through three stages: (1) incubation (green), where the pathogen is present and replicating but the patient is asymptomatic, (2) prodromal (blue), where the pathogen continues to replicate (white line) and causes non-specific symptoms due to immune activation, and (3) clinical illness (orange), where the symptoms of disease are the most severe. As the infection progresses through these stages treatment options often become more limited (red dashed line). Released miRNAs may reflect the stage of disease and provide important clinical information, particularly when the causative pathogen is difficult to detect.

The early detection of infectious disease is often critical to improve patient prognosis and limit the spread of disease. Treatment options often become increasingly limited as a disease proceeds (Wagstaff et al., 1994; McLean et al., 2015) (Figure 1). Rabies is a classic example of a disease where early intervention is crucial; it has an extremely long asymptomatic period of 2–3 months on average and once symptoms appear it is almost always fatal (WHO, 2019). Therefore, early intervention is key to survival.

Post-mortem brain smears are the current ‘gold standard’ diagnostic test for rabies infection, as the virus’ ability to evade the immune system and reside within neurons make standard molecular diagnostics difficult. Changes in miRNA profiles from rabies infected brain tissue have been documented and offer a promising insight into the miRNA response to lyssavirus infection (Zhao et al., 2012; Qi et al., 2014). However, there has only been one study examining the release of miRNAs into peripheral circulation due to lyssavirus infection. This study, completed in a mouse model, found seven miRNAs (miR-100, -187, -322, -706, -466, and -98) that were altered in the serum of infected mice compared to uninfected controls (Han et al., 2011). Low numbers of animals (only 3 control animals) and a heterogeneity in viral RNA and protein expression (measured via immunofluorescence, ELISA and nested RT-PCR) means that this work needs to be further validated. This hypothesis would also require testing in a model system that better replicates miRNA regulation in humans in order to translate miRNA profiling into a diagnostic tool for at-risk individuals.

Human immunodeficiency virus (HIV) is another well-known virus that would benefit from early detection. Infection with HIV causes systemic T-cell destruction and a corresponding reduction in cell mediated immunity (acquired immunodeficiency syndrome, AIDS) (Moir et al., 2011). It also results in damage to many organs through endothelial cell dysfunction and immune activation. As a result, there are numerous diseases associated with HIV infection, including viral and bacterial infections, cancers, encephalitis, gut disease, and cardiac damage (Lucas and Nelson, 2015). While the mortality rate from HIV-AIDS is decreasing, AIDS-related disease is still the leading cause of death in persons with HIV (Smith et al., 2014). The wide, and often devastating, impact of HIV infection has prompted numerous studies identifying miRNA biomarkers of both HIV infection and AIDS-related diseases.

Serum miR-21, -122, and -223 were found to distinguish HIV-positive from HIV-negative groups with a moderate (0.773, 0.726, and 0.804, respectively) receiver operating characteristic (ROC) area under the curve (AUC) (Thapa et al., 2014). A ROC AUC gives a succinct measure of the overall diagnostic accuracy of a test, with values typically ranging from 0.5 (no discrimination between groups) to 1 (perfect discrimination). For an in-depth review of ROC curves in evaluating diagnostic test accuracy, please see Hajian-Tilaki (2013). MicroRNA-3162-3p abundance in plasma from HIV patients was shown to differentiate new (<1 year post-infection) from old (>1 year) infections (Huang et al., 2018). Interestingly, a study by Yahyaei et al. (2016) found that miR-223 and -29a were elevated in individuals that were repeatedly exposed to HIV but did not contract a productive infection. These miRNAs may be purely markers of viral exposure or may play a key role in HIV resistance. Recently, a panel of four miRNAs (miR-16-5p, -20b-5p, -195-5p, and -223-3p) was developed and tested for HIV diagnostic utility (Biswas et al., 2019). Blind testing was able to identify all positive samples and healthy controls with 100% sensitivity and specificity. A different combination of four miRNAs (miR-16-5p, -206, -181c-3p, and let-7g-3p) were able to detect the eclipse phase of infection (the period of time between exposure and reliable diagnostic detection) with 100% sensitivity, 95.8% specificity, and a ROC AUC of 0.999. This highlights the use of miRNAs as disease biomarkers during the infectious period where conventional markers (antibodies, viral RNA/protein) are consistently undetectable (Biswas et al., 2019). Early therapeutic intervention in HIV infection has been shown to lead to long-term control of the infection with very low levels of persistent viremia (Nishimura et al., 2017).

Early detection can aid in quarantine, surveillance and biocontainment, factors that are especially relevant for diseases capable of being transmitted from animals to humans. Hendra virus (HeV) is a zoonotic paramyxovirus that can cause severe and often fatal infections in humans. The natural reservoir host for HeV is pteropid fruit bats, however, in 1994 it spilled over to horses and then humans (all with close contact to the infected horses) (Murray et al., 1995). Numerous outbreaks of HeV have occurred in Australia, mainly in horses, sparking concerns for human health. Stewart et al. (2013) identified elevated levels of miR-146a in the blood of experimentally challenged horses and ferrets (a model for human disease). This elevation was found to occur days before the detection of the viral genome. A subsequent study took an unbiased NGS-based approach to profile miRNAs within the blood of infected horses and found that 37 miRNAs were significantly altered in the blood of infected horses relative to healthy controls (Cowled et al., 2017). This study also examined the potential use of ratios of miRNAs, rather than individual abundances, to distinguish infected from uninfected animals. By doing so, inter-replicate variability was minimized, offering an alternative and possibly more robust method of identifying diagnostic miRNA signatures.

### Advantages of miRNA Biomarkers II: Improved Pathogen Identification

When symptoms of infectious diseases first appear, they are often non-specific (fever, malaise, headache, and lethargy) and provide little to no information about the causative agent. Consequently, clinicians may misdiagnose patients, which may have severe downstream consequences. One such example of this is viral encephalitis, where symptoms progress from an influenza-like illness to severe neurological abnormalities, including convulsion, speech loss, confusion, and coma. There are numerous neurotropic viruses that can cause viral encephalitis, ranging from common well-known agents, including herpes simplex virus (HSV), varicella-zoster virus (VSV), and enteroviruses, to deadly zoonotic pathogens such as Japanese encephalitis virus (JEV), lyssavirus (rabies), Nipah virus (NiV), and Hendra virus (HeV).

Although best known for being the causative agent of cold sores and genital warts, HSV is also the most common cause of viral encephalitis (Rabinstein, 2017). Serotype HSV-1, is one of the most intensively studied viruses, and is often used as the representative member of the alpha-herpesvirus subfamily. It was this intense scrutiny that led to the discovery of HSV-encoded miRNAs in 2006 by Cui et al. (2006). These miRNAs were found to be highly expressed during active infection (miR-H1) (Cui et al., 2006) and latency (miR-H2-6) (Umbach et al., 2008), and are actively packaged into exosomes and exported to uninfected cells (Kalamvoki et al., 2014). Despite no study outlining the use of these as diagnostic markers of HSV infection, these molecules have potential as biomarkers due to the specificity of these virally encoded miRNAs.

Recently, VSV has also been found to encode multiple small non-coding RNAs (Markus et al., 2017), however, these have not yet been found within the extracellular milieu. Clinically, VSV causes varicella (chickenpox) during the initial infection, but then becomes latent within dorsal root ganglion and cranial nerves and can reactivate to cause herpes zoster (shingles) (Gershon et al., 2015). A study Qi et al. (2014) found that several host miRNAs were altered in the serum of unvaccinated children with varicella compared to healthy controls. Five of these molecules, miR-197, -629, -363, -132, and -122, were found to differentiate VSV-infected individuals from controls, with a ROC AUC of 0.872. This combination of miRNAs could also distinguish the VSV-infected patients from others infected with similar pathogens, including pertussis (whooping cough), measles, and enteroviruses (Qi et al., 2014). Another study in 2016 found a further six miRNAs (miR-190b, -571, -1276, -1303, -943, and -661) that were differentially expressed in the serum of patients with reactivated herpes zoster (Li et al., 2016). Combined, these miRNAs could distinguish patients with herpes zoster from healthy controls and individuals infected with HSV, with a ROC AUC of 0.939 (Li et al., 2016).

Similarly, serum miRNA profiling of children with hand, foot and mouth disease (HFMD), caused by enteroviral infection, identified six miRNAs (miR-148a, -143, -324-3p, -628-3p, -140-5p, and -362-3p) that were able to discriminate between infected and healthy controls with a combined ROC AUC of 0.989, a sensitivity of 97.1% and a specificity of 92.7% (Cui et al., 2011). These miRNAs also displayed a unique profile between enterovirus and other microbial infections, including tuberculosis, pertussis, measles, mumps, and varicella. Interestingly, miR-140-5p was undetectable in healthy controls but was easily detectable in the serum of patients infected with any of the six pathogens investigated (Cui et al., 2011). Furthermore, five miRNAs (miR-148a, -143, -324-3p, -545, and -140-5p) were significantly altered between the two major causative agents of HFMD, coxsackievirus-16 and enterovirus-71, and demonstrated a moderate ability to distinguish between the two enteroviruses (combined ROC AUC of 0.761) (Cui et al., 2011). These results strengthen the hypothesis that miRNA biomarkers cannot only identify infected from uninfected individuals, but also distinguish between different causative agents. Further details on the potential of miRNAs in infectious disease diagnostics can be found in Ojha et al. (2019).

### Advantages of miRNA Biomarkers III: Detection of Latent Infections

Latency periods present significant hurdles in the current medical landscape. During these periods, the pathogen is present but cannot be detected via routine diagnostic methods. While the patient appears healthy, the pathogen can reactivate at any time, causing clinical disease or shedding infectious material. Tuberculosis (TB) is the leading infectious cause of death worldwide but healthcare efforts are often hampered by the large number of individuals with latent infections (WHO, 2018b). It is estimated that 23% of the global population have a latent TB infection, and 5–10% of these will develop active infection and clinical disease (WHO, 2018b). As there are currently no diagnostic platforms for latent TB, it is recommended to provide treatment to priority groups, including people living with HIV and children under 5 years old living with someone with confirmed TB (household contacts) (WHO, 2018b).

Several studies have identified circulating miRNAs in active pulmonary TB (Fu et al., 2011; Zhang et al., 2013; Cui et al., 2017; Barry et al., 2018; Pedersen et al., 2019). Recently, Lyu et al. (2019) examined exosomal miRNAs from the serum of patients with active and latent TB infections, as well as healthy controls. They found miRNA expression patterns unique to each group, including four miRNAs (let-7e-5p, let-7d-5p, miR-450a-5p, and miR-140-5p) that were elevated in latent TB infection when compared to both active infection and healthy controls (Lyu et al., 2019). Although their utility as diagnostic markers was not fully explored, they highlight the potential of miRNAs to identify latent TB infection. Circulating miRNAs have also been found to predict whether adults exposed to TB via infected household contacts would progress to active infection within the next 6 months (Duffy et al., 2018). These prognostic markers could be used to identify people at higher risk and better target the use of prophylactic treatments. At the opposite end of the TB infection timeline, researchers found four serum miRNAs that were able to identify patients who were cured of TB infection with an accuracy of 83.96% (Wang et al., 2018). Being able to discriminate between resolved and latent infections would provide clinicians with essential information to guide decisions for treatment and management of TB. COVID-19, another pulmonary disease where latency of up to 2 weeks and asymptomatic infections are a recognized problem (Jiang et al., 2020), is another disease where a more effective and accurate diagnostic is needed.

### Advantages of miRNA Biomarkers IV: Personalized Medicine

**FIGURE 2:**
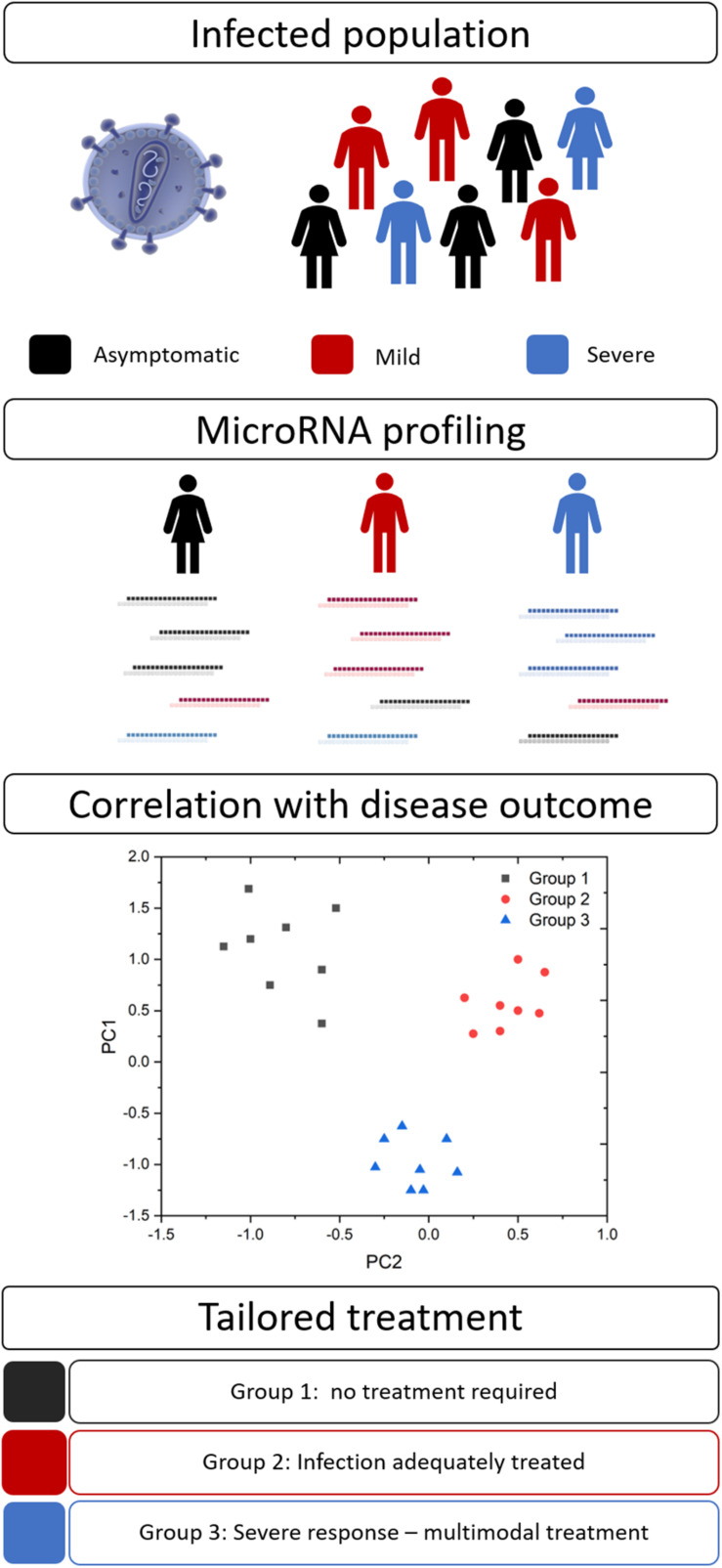
MicroRNA biomarkers in personalized medicine. Many infections result in a spectrum of disease severity, including asymptomatic (black), mild (red), and severe (blue) disease. Circulating miRNA signatures may differentiate between these groups and their ultimate disease outcome. This information would allow clinicians to tailor their approach and provide optimal therapeutic interventions.

Disease severity and the efficacy of anti-infective therapies can vary markedly between patients. Personalized medicine (also known as precision medicine or theranostics) recognizes that a ‘one size fits all’ approach is not always effective and attempts to tailor the treatment to achieve the best clinical outcomes. This healthcare concept is underpinned by biomarkers to provide pertinent information for timely and effective treatment decisions (Figure 2). Biomarkers in the field of personalized medicine often refer to an individual’s genotype, such as the BRCA mutation in ovarian cancer (Marchetti et al., 2018), however, this idea is being extended to other diagnostic or prognostic markers, such as miRNAs. As with many aspects of medicine, this approach first gained traction in oncology (Liu et al., 2014) but is now spreading to other areas, including infectious diseases.

Malaria is one such disease that results in a spectrum of severity, from asymptomatic to fatal infection. Recently, Burel et al. (2017) utilized a controlled human infection model to investigate the early molecular responses to malaria infection. They found that infected individuals could be stratified into two groups based on the changes in their circulating miRNA profile (low and high miR responders). Those patients in the high miR group were found to have increased CD4^+^ T-cell activation, a more robust antibody response, and reduced parasite burden (Burel et al., 2017). Importantly, the authors defined three miRNAs (miR-15a-3p, -30c-5p, and -30e-5p) that could distinguish these high and low-miR responders within days post-infection (Burel et al., 2017). These changes provide important information about the individual’s response to infection, helping to identify high-risk patients and guide treatment decisions. This divergent immune reaction may also explain the mixed success in malaria vaccine development (Rts, 2015).

Patients can also demonstrate a wide range of responses to therapeutic intervention, which can impact a clinician’s ability to provide an accurate prognosis. For example, 30–50% of individuals infected with hepatitis C virus (HCV) will not respond effectively to antiviral treatment (Cavalcante and Lyra, 2015). A liver-enriched miRNA, miR-122, has been shown to predict the efficacy of HCV therapy better than traditional circulating biomarkers, including alanine aminotransferase (ALT), albumin, and HCV RNA (Fan et al., 2017). Furthermore, circulating miR-122 was found to decrease alongside detectable HCV RNA during antiviral therapy, reflecting successful HCV treatment (Koberle et al., 2013). These results support the use of this miRNA to monitor the progression of HCV infection, predict treatment efficacy, and aid in prognostic accuracy.

Whilst much of this manuscript details the use of miRNA biomarkers in a diagnostic sense, the studies mentioned above highlight the potential of miRNAs to measure treatment efficacy, predict disease outcome and host responses, and guide optimal therapeutic selections as companion diagnostics.

## Caveats of miRNA Biomarkers

The development of host miRNAs as infectious disease biomarkers will require the successful navigation of several hurdles. Principle among these is the perceived lack of specificity among miRNAs responding to diverse infections. For example, miR-146a expression is known to fluctuate in response to hepatitis B virus (Wang et al., 2019), HCV (Abdel Motaleb et al., 2017), schistosomiasis (Cai et al., 2018), malaria (Cohen et al., 2018), JEV (Baluni et al., 2018), HeV (Stewart et al., 2013), and even prion disease (Bellingham et al., 2012). This miRNA has also been identified in non-infectious diseases, such as diabetes (Farr et al., 2015b) and heart disease (Quan et al., 2018). On its own, miR-146a has minimal disease specificity; indeed, it may be only indicative of a general immune response related to activation of the NF-κB transcription factor (Testa et al., 2017). However, this does not mean it should be disregarded, as the immune response may be an important marker of disease. Instead, miR-146a should be measured in conjunction with additional disease-specific miRNAs to ensure diagnostic accuracy.

Generalized miRNA responses to infection has important implications when trying to use these biomarkers to diagnose infections in the presence of co-morbidities or co-infections that may confound the results. Although there are studies that explore miRNA signatures in known co-infections, such as HIV/HCV (Anadol et al., 2015), this data is limited and is not able to take into account unknown confounding factors. Increasing biological replicates for miRNA biomarker discovery studies, as well as careful validation studies, will help to mitigate this issue and result in a more robust diagnostic signature.

Many studies identify potential miRNA biomarkers as those whose abundance changes in biofluids, utilizing a statistical cut-off to justify their selections. This approach, while effective in identifying large scale changes, does not automatically select for the most diagnostically relevant miRNAs. Instead, the degree of variation or the relationship of one miRNA to another may provide more effective differentiation between infected and non-infected individuals. Cowled et al. (2017) found that using ratios of two miRNAs ($\frac{m\,i\,R\,\_\,A}{m\,i\,R\,\_\,B}$) improved the adjusted *P*-values by normalizing for individual heterogeneity. Notably, these ratios were not arbitrarily selected; every possible combination of miRNAs was tested to identify those ratios that had the most statistically significant difference between groups. The use of miRNA ratios will have limited impact on their use in a clinical setting compared to the absolute or relative levels of a single marker. Indeed, they would be measured in concert (using a multiplex approach), the ratio calculated, and result presented based on whether they cross a pre-defined threshold.

This application of advanced bioinformatic analysis tools gives researchers more robust methods of identifying and evaluating predictive miRNAs. Feature selection algorithms (Saeys et al., 2007; Wang et al., 2016) allow computers to trawl through datasets highlighting the most predictive miRNAs, and have become more integral as researchers generate larger, more complex datasets. The introduction of machine learning into biological sciences has meant that researchers can create computational models that evaluate the predictive power of miRNA candidates on blinded samples. This approach can identify complex patterns and strengthen diagnostic classification. Machine learning has already been used to identify miRNA biomarkers for Ebola virus and *Mycobacterium tuberculosis* (Duy et al., 2016; Duffy et al., 2018) and will likely become more widespread. For this to occur, more investment needs to be made in advanced data analytics for biological sciences. In particular, microRNA biomarker research should move beyond simple differential expression analysis to capture the full spectrum of permutations and subtleties in expression of these molecules. Two factors that will greatly enhance successful outcomes from the use of computational analysis are: (1) an immediate focus on higher replicate numbers, and (2) proper use of holdout sets (validation samples), independent of samples used for initial biomarker discovery (training sets). These multivariate approaches (including the use of ratios) aid in the identification of composite miRNA signatures that can mitigate the lack of specificity seen in some individual markers.

Finally, despite advances in the rapidly advancing field of miRNA detection (Cheng et al., 2018; Kalogianni et al., 2018; Kilic et al., 2018), there are currently no industry standards for the detection and quantification of miRNAs for clinical diagnosis of disease. This handicap is due to the nature of miRNAs as diagnostic targets; they require isolation from biological fluids prior to detection; tend to exhibit subtle nucleotide differences leading to complications in probe specificity; and need to be protected from RNAse degradation during handling and processing (De Guire et al., 2013). In the future, emerging technologies for sample preparation and the detection of miRNAs for the diagnosis of infectious diseases should assist to improve current use of miRNAs as diagnostic markers.

## Current and Future Trends in miRNA Detection Systems

### Current miRNA Detection Platforms

Conventional approaches to the detection of miRNAs include northern blotting, microarrays, qPCR, and next generation sequencing (NGS). Contemporary miRNA biomarker studies tend to utilize qPCR and NGS, with northern blotting and microarrays falling out of favor due to limitations, including low sensitivity or specificity, and higher total RNA input requirements (often several micrograms for northern blotting). Recently, researchers have utilized Mass Spectrometry (MS) for the detection of miRs (Kullolli et al., 2014). Several reviews address qPCR and NGS for miRNA detection (Hardikar et al., 2014; Vincent et al., 2017).

To quantitate via qPCR, miRNAs are reverse-transcribed to cDNA and then amplified using defined primers. A sequence-specific probe, which contains a fluorophore and quencher, binds to the cDNA, and during amplification is cleaved by the endonuclease activity of the DNA polymerase. This releases the fluorophore and the resulting fluorescence is measured (Wong et al., 2015). It is a highly sensitive, highly specific gold standard against which to evaluate other detection systems. Despite its utility, qPCR suffers from several limitations, namely it is relatively expensive, and requires *a priori* defined primer and probe sequences. It is also relatively low-throughput, although this can be partially addressed through robotic handling systems, multiplexing and qPCR arrays (Wong et al., 2015).

Next generation sequencing also requires reverse-transcription and amplification but does so without using primers or probes specific to known miRNAs. It yields millions of short read sequences that can then be either analyzed *de novo* or mapped to a reference sequence such as a genome or miRNA sequence database (miRbase) (Kozomara et al., 2019). This technique allows quantitation of all miRNAs present in a sample, even novel sequences. Numerous NGS platforms are available with various proprietary chemistries but all are bulky, expensive (although prices are continuing to fall), time-consuming, and require complex sample preparation, instrument operation, and data analysis procedures. Despite this, it is the technique of choice for initial discovery of miRNA biomarkers and is typically followed by development of qPCR assays for further validation.

### New and Emerging miRNA Detection Platforms

Emerging technologies for the detection of miRNA biomarkers in infectious diseases aim to improve upon what is currently available, focusing on one or more of the following characteristics: portable, reliable, robust, rapid, specific, sensitive, low-cost, and user-friendly. Transitioning from costly and time-consuming lab-based methodologies to point-of-care (PoC) technologies is particularly important for the diagnosis and management of infectious disease outbreaks, where accurate and timely identification of infected individuals in resource-limited environments is critical for both patient care and disease containment.

**FIGURE 3:**
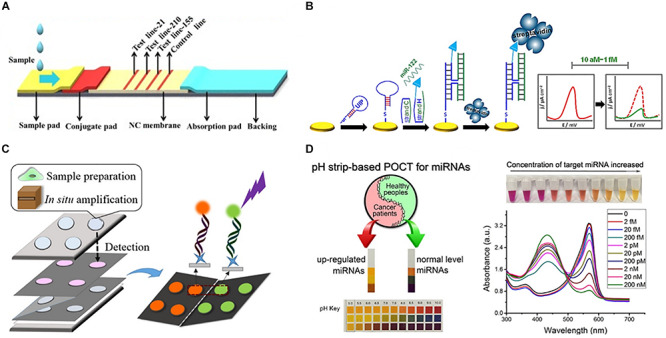
Representative approaches to the development of PoC miRNA sensing devices. **(A)** Lateral flow test device. Reprinted from Sensors and Actuators B: Chemical, volume 264, Zheng et al. (2018). Lateral flow test for visual detection of multiple MicroRNAs, Pages 320–326, Copyright (2018), with permission from Elsevier. **(B)** Electrochemical biosensor. Reprinted with permission from Labib et al. (2013a). Four-Way Junction Formation Promoting Ultrasensitive Electrochemical Detection of MicroRNA. Analytical Chemistry 85(20), 9,422–9,427. doi: 10.1021/ac402416z. Copyright (2013) American Chemical Society. **(C)** μPAD for miRNA extraction and quantitation. Reprinted with permission from Deng et al. (2017b). Paperfluidic Chip Device for Small RNA Extraction, Amplification, and Multiplexed Analysis. ACS applied materials interfaces 9(47), 41,151–41,158. doi: 10.1021/acsami.7b12637. Copyright (2017) American Chemical Society. **(D)** Colorimetric miRNA sensing strategy. Reprinted with permission from Feng et al. (2017). Detection of microRNA: a point-of-care testing method based on a pH-responsive and highly efficient isothermal amplification. Analytical chemistry 89(12), 6,631–6,636. doi: 10.1021/acs.analchem.7b00850. Copyright (2017) American Chemical Society.

As the current in-lab miRNA detection technologies continue to improve, the next question is how to translate this technology into PoC devices, and how to improve the current protocols in such a way that they are easily translatable. The predominant barrier to the development of PoC devices for miR detection is the low concentration of miRs in most biofluids. Whilst the concentration of individual miRs vary significantly in different biofluids, in healthy individuals, miRs in plasma are generally present in the fM range (9,000–134,000 copies/μL) (Mitchell et al., 2008). To overcome this, most examples of miRNA detection rely upon complex miRNA extraction protocols (such as multiple centrifugation or pipetting steps) or highly complex and bulky detection instrumentation (such as high-throughput NGS platforms or MS-based techniques) that are impractical for use in a resource limited PoC environment. To counteract this, many researchers have begun to develop new technologies that focus on miRNA extraction and detection in portable devices. An overview of these technologies can be found in Figure 3, while Table 1 compares their attributes. Whilst most of these examples focus on the diagnosis of cancer in humans, all of the technologies could be easily adapted to the diagnosis of infectious disease by modifying the target sequences of interest.

**TABLE 1:** Selected examples of emerging miRNA detection platforms.

**References**	**Detection**	**Sample**	**LOD**	**Multiplexed**	**Amplification**
	**Lateral flow**				
Gao et al., 2014	Visual/photographic	Cell lysate	60 pM	No	No
Deng et al., 2017a	Visual/cell phone/digital camera	Cell lysate	200 attomoles (10 pM)^a^	No	Target–recycled non-enzymatic amplification
Zheng et al., 2018	Visual/photographic	Human serum	7–85 pM (quantitative) 10–50 pM (visual)	3	No
	**Electrochemical**				
Cardoso et al., 2016	Electrochemical impedance spectroscopy (EIS) and square wave voltammetry (SWV)	Human serum	5.7 aM	No	No
Labib et al., 2013a	SWV	Human serum	2 aM	No	No
Labib et al., 2013b	SWV and EIS	Human serum	5 aM	No	No
Gai et al., 2018	Open circuit voltage	Human serum	2.7 aM	No	No
Cheng et al., 2015	SWV	Human serum	0.76 aM	No	No
	**μPAD**				
Deng et al., 2017b	Visual/cell phone/digital camera	Cell lysate	3 × 10^6^ copies (996 fM)^b^	2	Hairpin probe-exponential amplification reaction
Liang et al., 2017	Visual/fluorescence spectrometer	Cell lysate	30–60 aM	2	No
	**Colorimetric**				
Feng et al., 2017	Visual/UV-Vis	Cell lysate	100 fM (Visual) 9.3 fM (UV-Vis)	No	Netlike rolling circle amplification
	**Digital Microfluidic**				
Giuffrida et al., 2015	Fluorescence microscope	Cell lysate	3.3 attomoles (165 pM)	No	Molecular-beacon (MB)- assisted isothermal circular-strand-displacement polymerization
Shamsi et al., 2016	Electrochemiluminescence photomultiplier tube	Cell lysate	1.5 femtomoles (1.1 nM)	No	No

*^*a*^LOD concentration calculated from 20 μL sample volume from Deng et al., 2017a. ^*b*^LOC concentration calculated from 5 μL sample volume from Deng et al. (2017b).*

Lateral flow devices (strip tests) are useful tools for robust detection of biomarkers at the PoC due to their low-cost, simplicity, portability and specificity. In light of this, numerous groups have developed strip-based biosensors for miRNA detection (Hou et al., 2012; Gao et al., 2014; Deng et al., 2017a). Given most medical diagnoses are made after testing a panel of two or more biomarkers, Zheng et al. (2018) developed a multiplexed lateral flow assay for the simultaneous detection of miR-21, miR-155 and miR-210 in human serum samples at concentrations as low as 68, 7, and 17 pM, respectively, within 10 min (Figure 3A).

Deng et al. (2017a) sought to overcome the limited sensitivity of previous lateral flow based biosensors by incorporating a target recycling amplification strategy, whereby two sequence specific hairpins are used to amplify the signal without the need for added enzymes. The improved sensing mechanism required incubation at 37°C for 60 min and additional equipment including a handheld UV-light and mobile phone/digital camera but boasted a remarkable LOD of 200 attomoles from 20 μL of sample within 80 min.

Fully integrated patterned paper-based microfluidic devices (μPADs) have been developed featuring simple, user-friendly, robust and low-cost solutions for determination of a range of biomarkers using a variety of fabrication and detection strategies (Dungchai et al., 2009; Delaney et al., 2011; Dungchai et al., 2011). μPADs are particularly promising for the diagnosis of infectious diseases in resource limited settings as they are single-use, low-cost and can be readily disposed of by incineration; this negates the need for extensive sterilization procedures thus reducing the risks of accidental infection or contamination.

A variety of μPADs have been developed for PoC miRNA detection (Deng et al., 2017b) (Figure 3C). Deng et al. (2017b) inspired by previous examples of paper-based nucleic acid extraction (Connelly et al., 2015; Rodriguez et al., 2015), developed two paper based devices – one for miRNA extraction and amplification and a second for detection of the miRNA – which could be easily interfaced using magnetic sheets. The extraction and detection procedures required only a heating block for isothermal amplification, UV-light for quantum dot excitation and digital camera/mobile phone for fluorescence detection (Deng et al., 2017b). The μPAD exhibited a LOD of 3 × 10^6^ copies for two miRNAs (miR-21 and miR-155) from tumor cell lysate (Deng et al., 2017b). The simplicity, speed (90 min time-to-result) and low-cost of this paper-based device make it an ideal candidate for the diagnosis of infectious disease.

In spite of the application of μPADs for a variety of sensing applications, they are inherently limited in fluorescence-based sensing applications due to high background fluorescence and light scattering from the paper substrate (Pelton, 2009; Nery and Kubota, 2013). To overcome the challenges of conducting fluorescence assays on paper, Liang et al. (2017) grew a flower-like silver (FLS) layer on the paper substrate which decreased background fluorescence and produced ‘hot-spots’ where surface enhanced fluorescence of carbon nanodot labeled probes was observed. A detection limit of 30 and 60 aM was observed for miR-210 and miR-21, respectively, using the FLS μPAD. The device was also used for a qualitative visual pre-screening of analyte concentration due to a color change after the addition of hydrogen peroxide that caused oxidation of clear Ce^3+^ labels to orange Ce^4+^ (Das et al., 2007; Sardesai et al., 2013; Liang et al., 2017).

Visually identifiable color changes provide the simplest and lowest-cost detection strategy available. Inspired by the simplicity of the color-coded pH test strip Feng et al. (2017) exploited the production of hydrogen ions (H^+^) as a by-product of netlike rolling circle amplification (NRCA) to develop a pH dependent visual miRNA sensing methodology (Figure 3D). The group employed three pH indicators – cresol red, neutral red and m-cresol purple – and observed an LOD of 100 femtomolar (fM) for miR-21 by visual observation of the color change with the naked eye using a commercially available pH test strip or color indicator solution Where required, the samples could subsequently be analyzed via UV-Visible spectroscopy for quantitative sample analysis.

Whilst the majority of PoC sensing applications have focused on the development of colorimetric or fluorescence read-out strategies, these methodologies suffer from several drawbacks including high background signals which limit sensitivity. Numerous researchers have sought to overcome this drawback by employing less conventional detection strategies with interesting results.

Digital microfluidic (DMF) devices employ software-based electronic control of liquids and offer numerous advantages over conventional microfluidic systems as they eliminate the need for tubing and pumps whilst maximizing automation and reducing sample volumes (Choi et al., 2012; Giuffrida et al., 2015). Shamsi et al. (2016) employed electrochemiluminescence in a DMF platform for the detection of miR-143 to an LOD of 1.5 femtomoles within 40 min.

A number of research groups have successfully developed electrochemical biosensors for the sensitive and selective detection of miRNAs (Figure 3B). Herein, we will focus on representative examples which are particularly promising for PoC applications; a recent review by Gillespie et al. (2019) systematically addresses electrochemical microRNA sensing in further detail. Electrochemical detection strategies are readily adaptable to PoC applications due to the availability of low-cost portable potentiostats (Rowe et al., 2011; Doeven et al., 2015; Ainla et al., 2018) used in combination with low-cost commercially available screen printed electrodes or in-house produced alternatives (Dungchai et al., 2009, 2011).

Cardoso et al. (2016) employed electrochemical impedance spectroscopy for the determination of miR-155 in human serum at gold electrodes functionalized with a capture probe and obtained a detection limit of 5.7 aM from human serum samples. By developing a four-way junction electrochemical sensor, Labib et al. (2013a) were able to detect as little as 2 aM of miR-122 in human serum samples without PCR amplification. In a subsequent study, the researchers developed a three-mode duplexed sensor for the detection of two miRNAs – miR-32 and miR-122 – in human serum with a detection limit of 5 aM from 30 μL of sample (Labib et al., 2013b). Gai et al. (2018) further simplified conventional electrochemical sensing techniques by developing a two-electrode, self-powered biofuel-based sensor for the detection of miRNA at 2.7 aM. In an interesting approach combining electrochemical sensing with cadmium modified titanium phosphate nanospheres, Cheng et al. (2015) developed a miRNA-21 sensor with a detection limit of 0.76 aM that could be applied for the direct analysis of human serum samples (Figure 3B).

## Validation and Translation of miRNA Diagnostics

**FIGURE 4:**
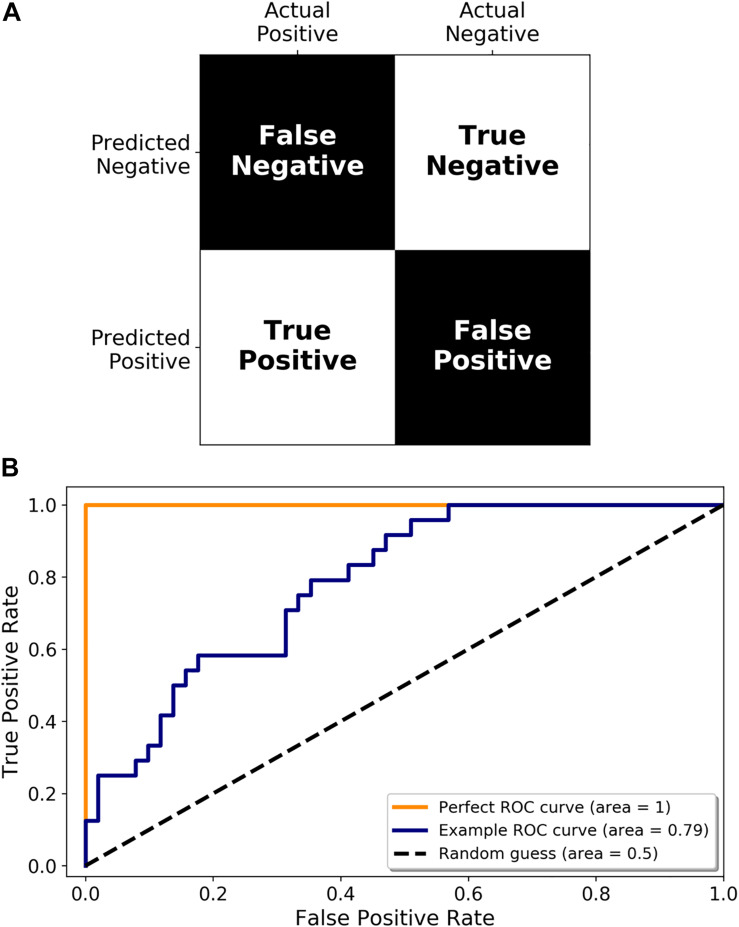
Metrics for diagnostic test validation. **(A)** Example confusion matrix from which the common metrics (accuracy, sensitivity, and specificity) can be calculated. **(B)** ROC graph showing a perfect ROC curve (orange line), and example ROC curve (blue line), and the diagonal indicating a random classification model (random guess and black dotted line).

Once a set of miRNAs have been identified as potential disease biomarkers, their use in a diagnostic assay needs to be adequately assessed. Accuracy is an oft-cited metric but may not always be appropriate (for example, if you expect that 99% of the samples you test are going to be negative, then a test that simply labels all samples as negative will be 99% accurate). There are a multitude of other metrics available, including sensitivity (proportion of infected samples who tested positive), and specificity (proportion of non-infected samples who tested negative), all of which are simply different ways of interpreting a confusion matrix (predicted positive or negative classification vs. the true classification, see Figure 4A). Another important performance metric, which has been referenced in some studies, is the Receiver Operating Characteristic (ROC) curve. A ROC curve is a graphical representation of how the true positive rate (TPR) and false positive rate (FPR) change as the classification threshold is varied between the infected and non-infected groups. The area under the curve (AUC) is also often calculated, giving a succinct measure of the overall performance of the test. If a diagnostic test has zero predictive power, the TPR and FPR will be equal, resulting in a diagonal line and an AUC of 0.5 (Figure 4B, Random guess). Alternatively, if it correctly identifies all samples 100% of the time, then the ROC curve, the TPR is equal to 1 and the FPR is equal to 0, resulting in an AUC of 1.0 (Figure 4B, Perfect ROC curve). In reality, a diagnostic test usually falls somewhere between these two possibilities (Figure 4B, Example ROC curve). A ROC curve can also help choose the classification threshold. There is a fine balance between the TPR and FPR and the threshold that is chosen is dependent on the ultimate purpose of the test. For example, if a patient is exposed to rabies, it is more acceptable to be falsely identified as infected, and therefore treated, than falsely identified as not infected, sent home, and ultimately succumb to infection. Therefore, a miRNA diagnostic test for rabies infection would heavily favor the detection of true positives (sensitivity) even if that meant it detected a larger number of false positives. It is imperative that all potential diagnostics be evaluated in the context of their use.

Moreover, when translating a miRNA biomarker signature to a diagnostic device, it is pertinent to have a clear understanding of how the end consumer will use the test, and how to maximize its utility. Many current diagnostic tests, such as serology, need to be sent to a diagnostic laboratory to be analyzed. For infections that are time-critical this is a costly delay, hence the time it takes to generate a result is an important consideration. Some diagnostic platforms may be used for initial screening or used in rural or remote areas where access to a laboratory is limited. PoC devices may alleviate these constraints as samples do not need to be transported to a diagnostic laboratory. In other circumstances a test undertaken in an accredited diagnostic laboratory may be more desirable. Regardless of which format the test is ultimately configured, care needs to be taken to ensure it is fit-for-purpose.

A key step toward final test adoption is evaluation in clinical trials to define test performance. Tests may be scrutinized at: (1) a prototype evaluation (phase I), (2) an evaluation under ideal conditions (phase II), or (3) an evaluation under real-life conditions (phase III). At a minimum, such evaluations should provide information not only on a test’s diagnostic accuracy but also on its repeatability, reproducibility, and ease of use. Clinical trials require biomarkers to be assessed against a gold standard or reference method, often selected in consultation with regulators during trial design. Clearly, undertaking clinical trials requires significant financial resources to enroll patients, disease experts and clinicians, process large sample numbers and collect clinical data. For some diseases, particularly emerging infectious diseases, this cost may be viewed as prohibitive given the market opportunity and expected return on investment. Gaining access to appropriate samples or clinical expertise also may present a challenge. The cost of clinical trials will also be significantly impacted by scope. For instance, some would argue that in order to demonstrate that a biomarker-based diagnostic test improves patient outcomes over an existing or predicate test, a randomized controlled trial should be conducted. Here, randomized comparisons are made between two diagnostic interventions (one reference and one experimental) with identical therapeutic interventions based on the results of the competing diagnostic tests. Study outcomes are clinically important consequences of diagnostic accuracy. While considered the gold standard, randomized controlled trials are rarely cited due to their significant costs and a lack of regulatory pressure.

In addition to clinical trials, navigation through the regulatory approval process is a key step toward adoption of biomarker-based diagnostics. The requirements for licensing diagnostic tests are complex and differs considerably from country to country, with many lacking regulatory procedures to assess the safety, quality or effectiveness of *in vitro* diagnostic tests (IVDs). In the United States, the Food and Drug Administration (FDA) regulates IVDs with the intended use of the device determining how much data, type of data, and approval route to use. Within Europe the European Medicine Association (EMA) fulfills this role. Whether or not the biomarker test is substantially equivalent to an existing test, and the context in which a test will be used (critical versus low or moderate risk) are factors in the level of regulation and data required. Additionally, the sample type and route of collection will impact the ease of use and clinical uptake of an IVD. Less invasive samples, such as saliva or sputum are often preferred over blood or sera. Indeed, miRNAs have been found in almost all biofluids, including saliva (Min et al., 2018), urine (Ben-Dov et al., 2014), breast milk (Kelleher et al., 2019), and tears (Kim et al., 2019), however, the use of these less invasive sample types would depend on the underlying disease. For example, salivary miRNAs may be useful for the diagnosis of mouth infections (Min et al., 2018) but may not be useful for infections in other areas of the body. Blood, serum, and plasma are often used for biomarker identification as peripheral samples often contain markers of localized disease. Consideration for disease pathology and clinical practice must be taken when looking to translate miRNA biomarkers.

While the market for miRNA biomarkers is still in its infancy, some products (primarily in oncology) are currently available to clinicians, while others are entering pre-clinical and clinical trials (Bonneau et al., 2019). Interpace Diagnostics is one notable example, with their product ThyraMIR^®^ (a 10 miRNA panel) distinguishing between benign and malignant thyroid cancer. This test is combined with an oncogene NGS panel (ThyGeNEXT^®^) to stratify patients on whether they require surgery and if so, the extent of the surgical intervention. This approach, first detailed in 2015 (Labourier et al., 2015), has since been refined and demonstrates significant clinical utility in stratifying patients based on their risk of malignancy after 9 months (Sistrunk et al., 2020). Interpace Diagnostics has also been successful in obtaining coverage for these tests by some of the largest health insurance providers in the United States, a significant step in clinical adoption. Case studies such as this can provide invaluable insight into the journey of miRNA biomarkers from discovery to clinical application.

## Conclusion

For many infectious diseases, the current diagnostic technologies are inadequate. Many rely on the presence of symptoms or pathogen-specific antibodies or cannot be utilized until the pathogen has replicated to a detectable titer. New biomarkers are sorely needed to underpin the next generation of diagnostic platforms. MicroRNAs have substantial potential as biomarkers of infection, as evident in the growing body of research data. These molecules are altered in biofluids due to infections with bacteria, parasites, viruses, and even prions. Their use as diagnostic or prognostic markers is not without its challenges, however, with robust analytical and validation methods, their use may alleviate the shortcomings of current tests, leading to improved patient outcomes. In conjunction with the identification of sufficiently predictive miRNAs, the exciting progress in miRNA sensing technologies and the emergence of recent, commercially available miRNA-based diagnostics, means that miRNAs will inevitably be employed as powerful new tools in diagnostic, prognostic and even therapeutic strategies for infectious diseases.

## Author Contributions

All authors agreed on the final draft of the manuscript prior to submission. LT, EK, and RF wrote the first draft and revised the manuscript. CC, CS, MD, and AB reviewed and edited the manuscript.

## Conflict of Interest

The authors declare that the research was conducted in the absence of any commercial or financial relationships that could be construed as a potential conflict of interest.

## Footnotes

1 http://www.mirbase.org/
